# Genome-wide association analysis of chickpea germplasms differing for salinity tolerance based on DArTseq markers

**DOI:** 10.1371/journal.pone.0260709

**Published:** 2021-12-01

**Authors:** Shaimaa Mahmoud Ahmed, Alsamman Mahmoud Alsamman, Abdulqader Jighly, Mohamed Hassan Mubarak, Khaled Al-Shamaa, Tawffiq Istanbuli, Osama Ahmed Momtaz, Achraf El Allali, Aladdin Hamwieh

**Affiliations:** 1 Gene expression and regulation technology lab, Department of Plant Molecular Biology, Agricultural Genetic Engineering Research Institute, Agricultural Research Centre, Giza, Egypt; 2 Department of Biodiversity and Crop Improvement, International Center for Agriculture Research in the Dry Areas, Agricultural Research Centre, Giza, Egypt; 3 Molecular Genetics and Genome Mapping, Agricultural Genetic Engineering Research Institute, Giza, Egypt; 4 African Genome Center, Mohammed VI Polytechnic University, Ben Guerir, Morocco; 5 Agriculture Victoria, AgriBio, Centre for AgriBiosciences, Bundoora, VIC, Australia; 6 Plant Production Department, Faculty of Environmental Agricultural Sciences, El-Arish, North Sinai, Egypt; 7 Geoinformatics & Research Data Management, International Center for Agriculture Research in the Dry Areas, Giza, Egypt; Mahatma Phule Krishi Vidyapeeth College of Agriculture, INDIA

## Abstract

Soil salinity is significant abiotic stress that severely limits global crop production. Chickpea (*Cicer arietinum* L.) is an important grain legume that plays a substantial role in nutritional food security, especially in the developing world. This study used a chickpea population collected from the International Center for Agricultural Research in the Dry Area (ICARDA) genebank using the focused identification of germplasm strategy. The germplasm included 186 genotypes with broad Asian and African origins and genotyped with 1856 DArTseq markers. We conducted phenotyping for salinity in the field (Arish, Sinai, Egypt) and greenhouse hydroponic experiments at 100 mM NaCl concentration. Based on the performance in both hydroponic and field experiments, we identified seven genotypes from Azerbaijan and Pakistan (IGs: 70782, 70430, 70764, 117703, 6057, 8447, and 70249) as potential sources for high salinity tolerance. Multi-trait genome-wide association analysis (mtGWAS) detected one locus on chromosome Ca4 at 10618070 bp associated with salinity tolerance under hydroponic and field conditions. In addition, we located another locus specific to the hydroponic system on chromosome Ca2 at 30537619 bp. Gene annotation analysis revealed the location of rs5825813 within the Embryogenesis-associated protein (*EMB8-like*), while the location of rs5825939 is within the Ribosomal Protein Large P0 (*RPLP0*). Utilizing such markers in practical breeding programs can effectively improve the adaptability of current chickpea cultivars in saline soil. Moreover, researchers can use our markers to facilitate the incorporation of new genes into commercial cultivars.

## Introduction

The global food demands are exponentially expanding, while water scarcity will affect 1.8 billion people by 2025. Salinity is one of the most crucial problems facing food security [1]. According to FAO, over 6.5% of the world’s land is affected, translating into 800 million HA of arable lands and expanding dramatically [2]. Filling the gap between consumption and production requires more research to enhance unprepared commercial varieties to face environmental changes and improve their tolerance. Chickpea is a legume crop that is overly sensitive to salinity and severely impacts grain yield [3]. Upon exposure to salt stress, the meristems accumulate salts in the vacuoles of the xylem to lower their osmotic potential till reaching high concentrations [4]. Research report that developing new cultivars with high salinity tolerance is the most economical strategy to improve cultivar’s adaptability to salt stress [5, 6].

Genebanks around the world hold a massive number of genotypes with high unexplored potential. The Focused Identification of Germplasm Strategy (FIGS) improves the efficiency of the initial selection from genebanks for specific adaptive traits. The method predicts the accession’s potential, assuming that the environmental selection pressures, where these genotypes were initially collected, will be reflected on them [7]. FIGS uses both trait and environmental data to define a set of genotypes with a high probability of containing the desired traits based on quantifying the trait-environment relationship [8]. FIGS has been successfully used to screen new genes related to abiotic and biotic stresses in different plant species [9]. The massive growth in phenotypic and genotypic assessment technologies has encouraged dissecting genomic loci responsible for crop saline tolerance. Due to their abundance in genomes, evolutionary relationship, suitability for genetic diversity analysis, and association with complex phenotypic traits, single nucleotide polymorphism (SNP) markers have gained remarkable value in plant molecular genetics [10].

Genome-wide association study (GWAS) through SNP genotyping has a significant impact on identifying genetic regions associated with quantitative and complex traits. Many methods have been developed to reduce genome complexity. The diversity array technology (DArT) assay provides a remarkable advantage via an inventive selection of genome fractions corresponding predominantly to active genes (http://www.diversityarrays.com/dart-application-dartseq).

Few research articles reported the localization of salinity tolerance-related loci in chickpea. For example, Pushpavalli et al. [11] reported two key genomic regions on Ca5 and Ca7 that harbor QTLs for six and five different salinity tolerance associated traits, respectively. Based on gene ontology annotation, the authors roughly identified 48 putative candidate genes responsive to salinity stress; 31 genes on CaLG05 and 17 genes on CaLG07. Most genes were known to be involved in achieving osmoregulation under stress conditions. In addition, other researchers used differential expression gene analysis to detect abiotic stress-related genes that were significantly up-regulated in the tolerant genotypes and down-regulated in the sensitive genotypes under salt stress [5].

In the present study, we screened a chickpea population with diverse origins for salinity tolerance. We genotyped the germplasm with DArTseq markers, which were used to detect loci associated with salinity tolerance in chickpea through multi-trait GWAS (mtGWAS). In addition, we explored the population structure and phylogenetic diversity of the studied chickpea genotypes to evaluate their genetic background.

## Materials and methods

### Materials

The studied germplasm panel (drought subset) covers 186 different genotypes collected from 28 provinces in 13 countries across the globe. We selected the drought subset from ICARDA’s genebank using the Focused Identification Germplasms Strategy (FIGS) [7](S1 Table). This subset includes 152 and 34 genotypes of Kabuli and Dezi genotypes, respectively (S1 Table). Most of these genotypes originated from several saline areas in Pakistan and India.

### Phenotyping salinity tolerance

We performed the experiments in a field located in the Arish province, Sinai, Egypt, and in a greenhouse at the Agricultural Research Center (ARC) in Giza, Egypt using the hydroponic system. Using field and greenhouse experiments, we evaluated chickpea salinity tolerance during 2014 and 2015. The experiments were conducted in two replications using the Alpha Lattice design. We used a dripping water irrigation system in the field and irrigated it once every two weeks. A soil sample was air-dried, softened, and sieved before preparing soil pots to determine the salt concentration in the soil of the fields. Then soil solution was extracted to determine pH and the concentration of cations and anions [12]. Additionally, we analyzed the water used for field irrigation. The salt concentration in the field was 344 ppm in the first 30 cm depth, 904 ppm in depths from 30 to 60 cm, and 848 ppm in the depth of more than 60 cm. The water irrigation analysis revealed that the average salt concentration was 897 mM, and the pH was 7.2. In the greenhouse, three seeds from each accession were germinated in small pots (5 cm) containing a mixture of peat moss (40%) and perlite (60%). After two weeks, we transferred the pots to large tanks (150 cm X 230 cm) containing saltwater solution to induce salt stress. Half the strength of the Hogland solution has been added to the solution. We used the electrical conductivity (EC) meter (Hanna HI8733) to measure and fix the salt concentration at 100 mM. The pH was measured daily and adjusted at a level of 8. The Salinity stress score was measured as the necrosis score. We conducted the scoring when the necrotic symptoms appeared on the seedlings one week after salt treatment. The phenotypic scaling of salinity tolerance rate (STR) ranged from 1 to 5 (1: plants with normal healthy leaves. 2: one-third or fewer leaves showed chlorotic symptoms. 3: half or fewer leaves showed chlorotic symptoms. 4: two-third or more leaves showed chlorotic symptoms, or only upside leaves survived. 5: plants completely dead). The plants were scored four times during the experiment over a 2-week interval.

### DNA extraction

We extracted DNA from young leaves of seedlings that are 4- to 6-week-old before salt treatment using the cetyltrimethylammonium bromide (CTAB) method, as described by Rogers and Bendich (1985). In brief, fresh leaf material from seedlings was dried and grounded into a fine powder. Subsequently, we added the powder to a 2 mL Eppendorf tube with 1 mL pre-warmed 2X CTAB buffer 2% CTAB, 0.1 M Tris HCl (pH 8.0), 1.4 M NaCl, 20 mM Ethylenediaminetetraacetic acid (EDTA). The suspension was mixed and incubated at 65°C for 30 min. The suspension was cooled at room temperature (RT) for 5 min, 1 mL chloroform-isoamyl alcohol (24:1) was added to the tube, and the suspension was gently mixed by shaking for 10 min. The suspension was centrifuged at 4,500 rpm (Beckmann YA-12) for 20 min at RT, and the supernatant was transferred to a new tube. The DNA was precipitated with 1mL of cold isopropanol. The DNA was transferred into a micro-centrifuge tube and washed twice with a washing buffer (70% ethanol and 200 mM sodium acetate) for 20 min. After air-drying for about 10–20 min, the DNA was dissolved in 200 *μ* of 1 X TE buffer 10 mM Tris HCl, pH 8.0, 1 mM EDTA.

### Molecular marker analysis

We used the Diversity Arrays Technology (DArT^®^) and SNP markers panel for genotyping the chickpea population with high-density. Out of the collected 186 different genotypes, genotypes were sent for DArT and SNP, using 50 μl of a 100 ng μl/1 DNA of each sample. We sent DNA to Triticarte Pty. Ltd. Australia (http://www.triticarte.com.au) for marker genotyping (Chickpea DArTseq and SNP panel version 1.0) as a provider for commercial service. We retrieved 11979 markers (5305 SNPs and 6674 DArT markers) (Table 1 and S2 Table). Out of these markers, 1856 polymorphic marker loci with call rates both greater than 80%, minor allele frequency (MAF) ≥ 5%, and heterozygosity ≤ 15% were selected for genome-wide association analysis. We used the BLAST tool [13] was used to assign SNP and DArT markers to chickpea chromosomes [14].

**Table 1:** The Chromosomal distribution of the 1854 SNP and DArT markers used in this study.

Chromosome	SNP	DArT	Total
Ca1	108	101	209
Ca2	66	70	136
Ca3	70	61	131
Ca4	256	316	572
Ca5	40	45	85
Ca6	89	112	201
Ca7	91	110	201
Ca8	31	33	64
CaN	67	188	255
Total	818	1036	1854

### Data analysis

Best linear unbiased estimation (BLUE) was calculated for each phenotypic score by adjusting for spatial effects by fitting column, row, and replicate effects in a linear mixed model fitted using ASReml-R [15]. The genotype by genotype × environment interaction (GGE) biplot analysis was conducted using the R package GGE with the default parameters (http://kwstat.github.io/gge/). ADMIXTURE software [16] was used to infer population structure with the number of underlying subpopulations (K) ranging between 2 and 20. We run the analysis with 100 random replicates and 20 cross-validations. The most probable K was determined when the average cross-validation values across the 100-replicate started to increase. Pairwise linkage disequilibrium (LD) between markers within the same chromosome was estimated with the r^2^ statistics [17].

The r^2^ values were plotted against the physical distance among markers, and the second LOESS decay curve was fitted to determine the size of LD blocks. Using computationally intensive analysis, multi-trait GWAS was used to improve the accuracy of the GWAS results. Compared to comparable univariate approaches, multi-trait GWAS methods produce a higher true-positive quantitative trait nucleotide detection rate across all tested simulation settings while reducing false positives due to population structure and kinship [18]. Multi-trait GWAS was fitted using GEMMA software [19] based on all hydroponic records and both hydroponic and field records together with the default parameters. The genomic relatedness matrix was used as a covariate to control for population stratification [20]. We calculated the independent number of markers using the Type I Error Calculator (GEC) method to determine the significance threshold considering multiple testing using several markers [21]. Applying such a method in crops is important to avoid the stringency of methods such as Bonferroni that assumes independent tests, given that markers are dependent due to linkage disequilibrium.

We explored the genotypic richness and genotypic evenness using several diversity indices. These indices include lambda Simpson’s Index [22], Hexp Nei’s unbiased gene diversity [23], the index of association (I^a^) [24], the standardized index of association (rbarD), principal component analysis (PCA), and analysis of molecular variance (AMOVA). The calculations were done using adegenet R package [25]. The AMOVA analysis was carried out using country of origin and population information generated from population structure analysis. A dendrogram was constructed with the Unweighted Pair Group Method with Arithmetic means (UPGMA) using Power marker software [26]. Phylogenetic trees were constructed using iToL [27]. We run the Mantel test to study the relationship between SNP and DArT markers using the GenAlex molecular analysis tool [28]. Nine thousand nine hundred ninety-nine random iterations were used during the analysis to calculate correlation matrices between the SNP and DArT markers.

## Results

### The effect of salinity stress on chickpea genotypes

**Fig 1:**
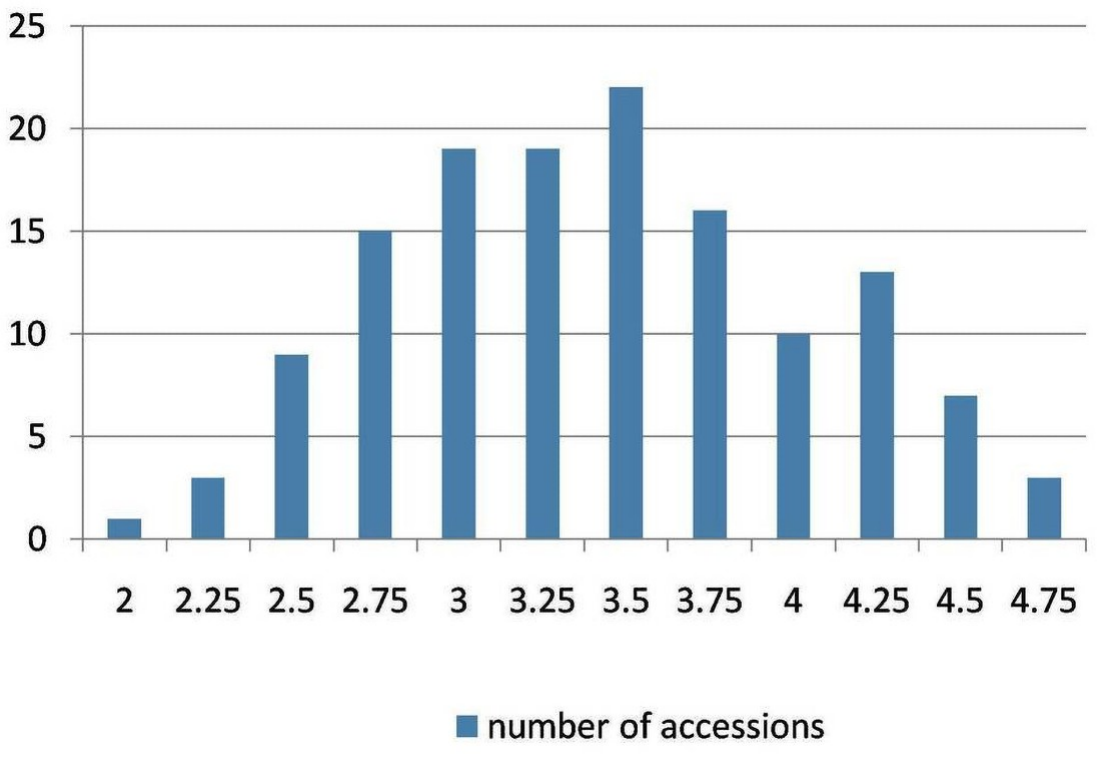
The salinity tolerance in the studied chickpea. The distribution for salinity tolerance phenotypic scaling among different chickpea genotypes.

The genotypes showed variable tolerance levels to salinity (Fig 1). Out of 186 genotypes used in this study, only forty-seven (25.3%) genotypes were observed to be tolerant (STR score ≤ 2.5). The salinity tolerance was normally distributed among chickpea genotypes. Based on the hydroponic and field experiments average, seven tolerant genotypes IGs (70782, 70430, 70764, 117703, 6057, 8447, and 70249) were identified with STR ≤ 2.5, of which six were from Pakistan and only one from Azerbaijan (Table 2). The first two principal components explained 76% of the total variation together. Several genotypes were clustered close to the center of the plot, indicating their stability in field and greenhouse treatments.

**Table 2:** The best genotypes and their origin were identified based on the mean of the salinity tolerant rate (STR) in greenhouse and field.

Best genotype	Field	Hydroponic	Origin	Province
IG70782	2	2	Pakistan	Punjab
IG70430	2.5	2	Pakistan	Punjab
IG70764	2.5	2	Pakistan	Sindh
IG117703	2.5	2	Pakistan	Punjab
IG6057	2.5	2.5	Pakistan	NWF
IG8447	2.5	2.5	Azrabejan	Lankaran
IG70249	2.5	2.5	Pakistan	Sindh

### Relatedness, population structure, and linkage disequilibrium analyses

**Fig 2:**
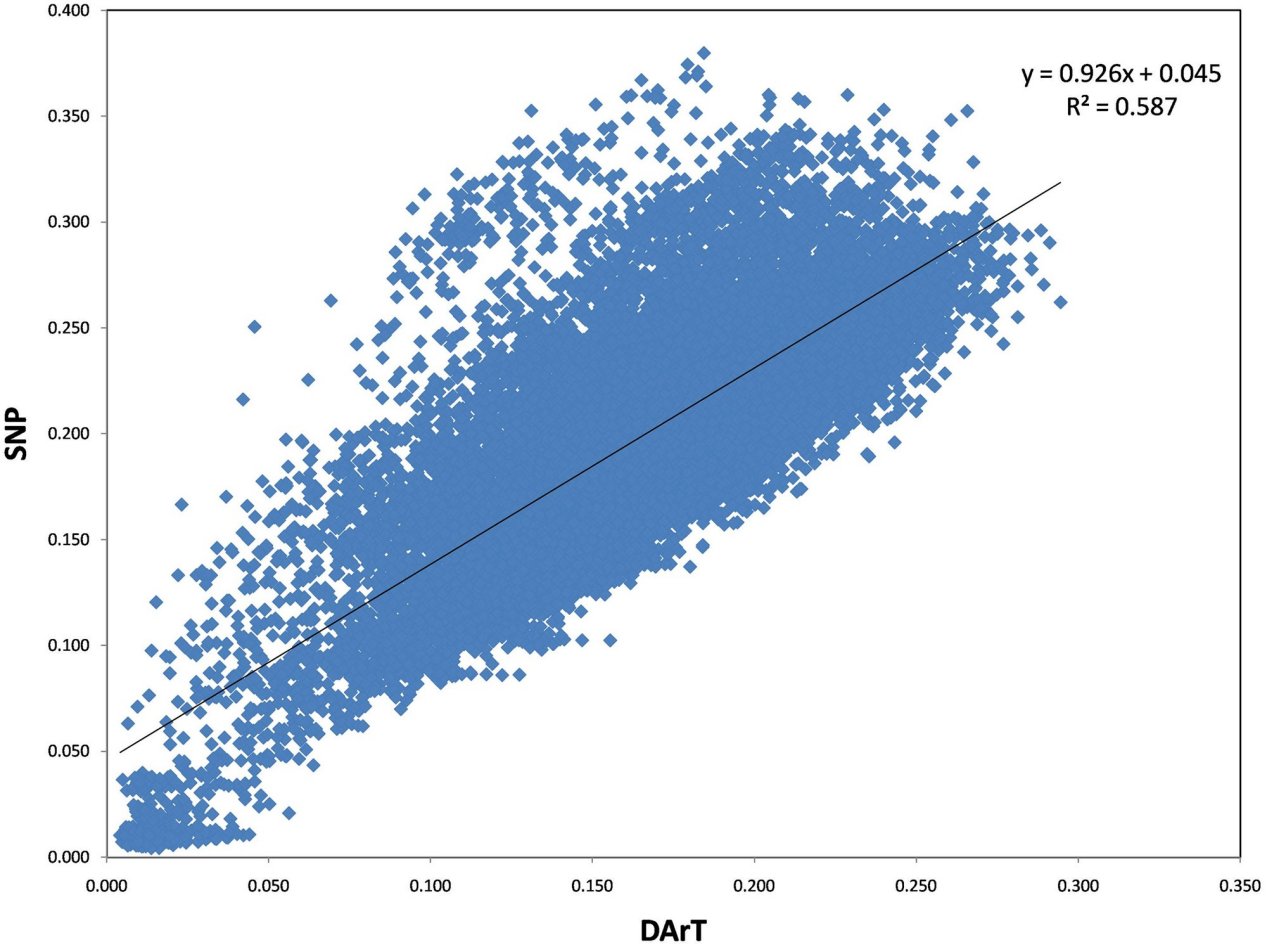
The correlation analysis of the genetic markers. Correlation analysis of genetic data obtained through SNP and DArT technologies.

**Fig 3:**
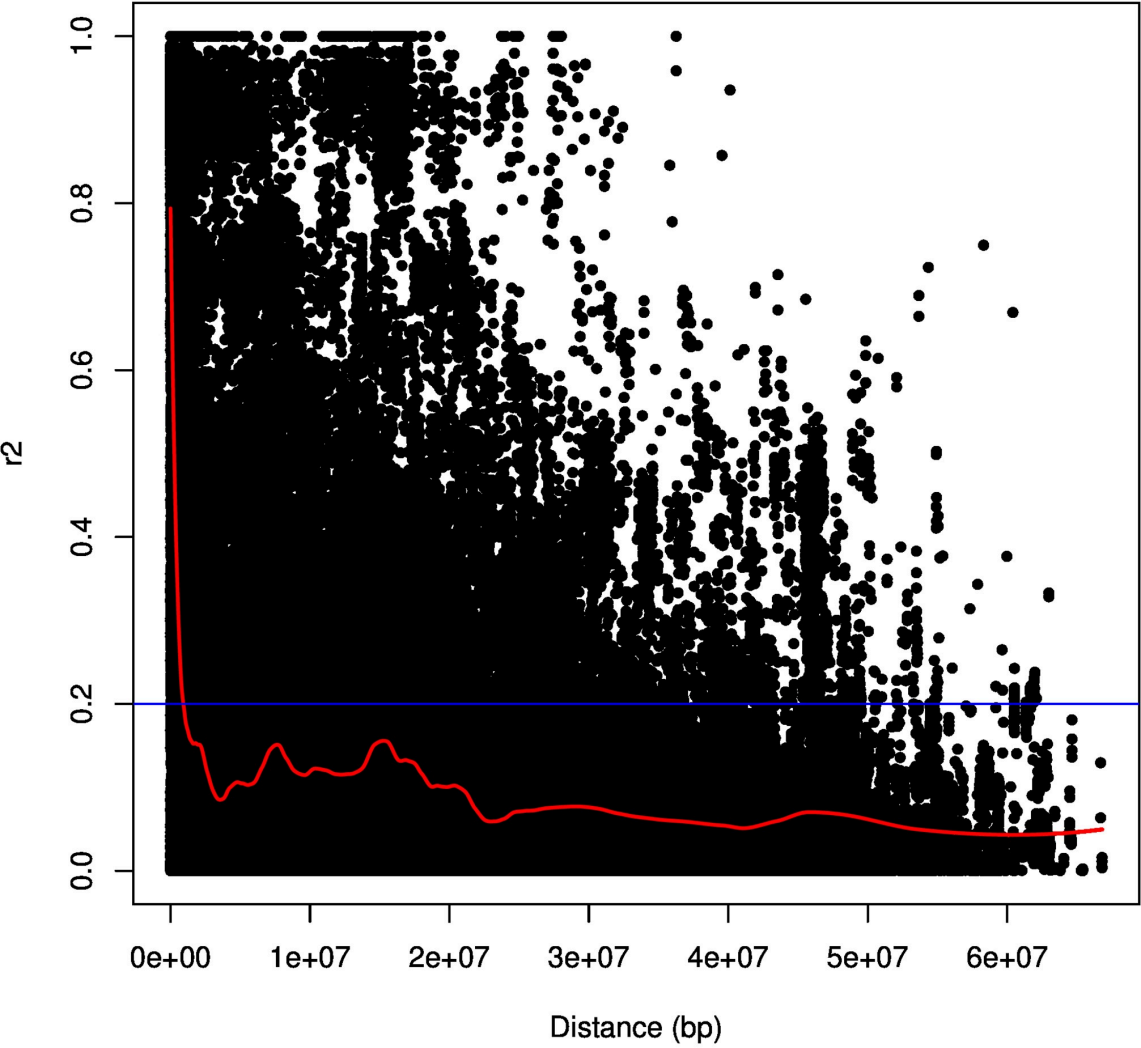
The linkage disequilibrium decay analysis. The linkage disequilibrium decay analysis showing the r^2^ values of the 1854 SNP and DArT markers used in this study.

The polymorphism information content (PIC) values for DArT and SNP markers range from 0.01 to 0.375 and 0.01 to 0.58, respectively. The latter implies moderate loci informativeness for SNP and DArT markers. Filtering the markers for MAF (Marker Allele Frequency), heterozygosity, and call rate resulted in a total of 1856 high-quality markers for downstream analyses. Given the size of the chickpea genome (738 MB), our marker density can be calculated as one marker every 738/1854 = 0.4 MB. These markers were distributed across the chickpea genome, where Ca4 has 572 markers followed by 209 markers in Ca1 (Table 1). The genetic data retrieved from the studied chickpea population was used to study the statistical correlation between DArT and SNP markers. The Mantel test analysis revealed a 0.5 R^2^ positive correlation between SNP and DArT markers (Fig 2). The linkage disequilibrium decay analysis showed that the r^2^ values started to decay below 0.2 at around 0.9 Mb, indicating that we have enough marker coverage along the genome (Fig 3).

**Fig 4:**
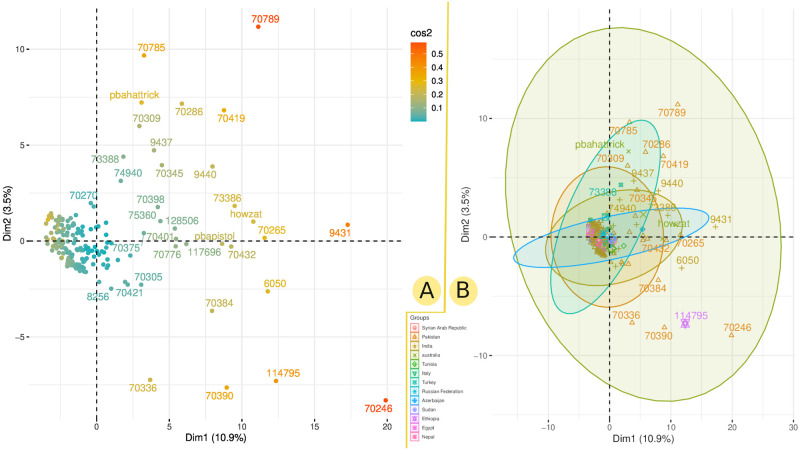
The PCA analysis generated by genetic markers. The PCA analysis of the studied chickpea population shows the contribution of chickpea genotypes based on *cos^2^* value (A) and their grouping versus country of origin (B).

By transforming initial variables into a smaller set of variables, PCA reduces the dimensionality of the data. The PCA analysis was carried out using the genetic and geographical information from the chickpea population under study (Fig 4). The squared cosine value (*cos^2^*) for individual factors determined that several chickpea genotypes strongly contributed to PCA, with genotypes from India and Pakistan being the most prominent genotypes (Fig 4A). The observed variation for the first, second, and third principal components, according to eigenvector analysis, was about 15.13%, 4.8%, and 4.3%, respectively (Fig 4B).

**Fig 5:**
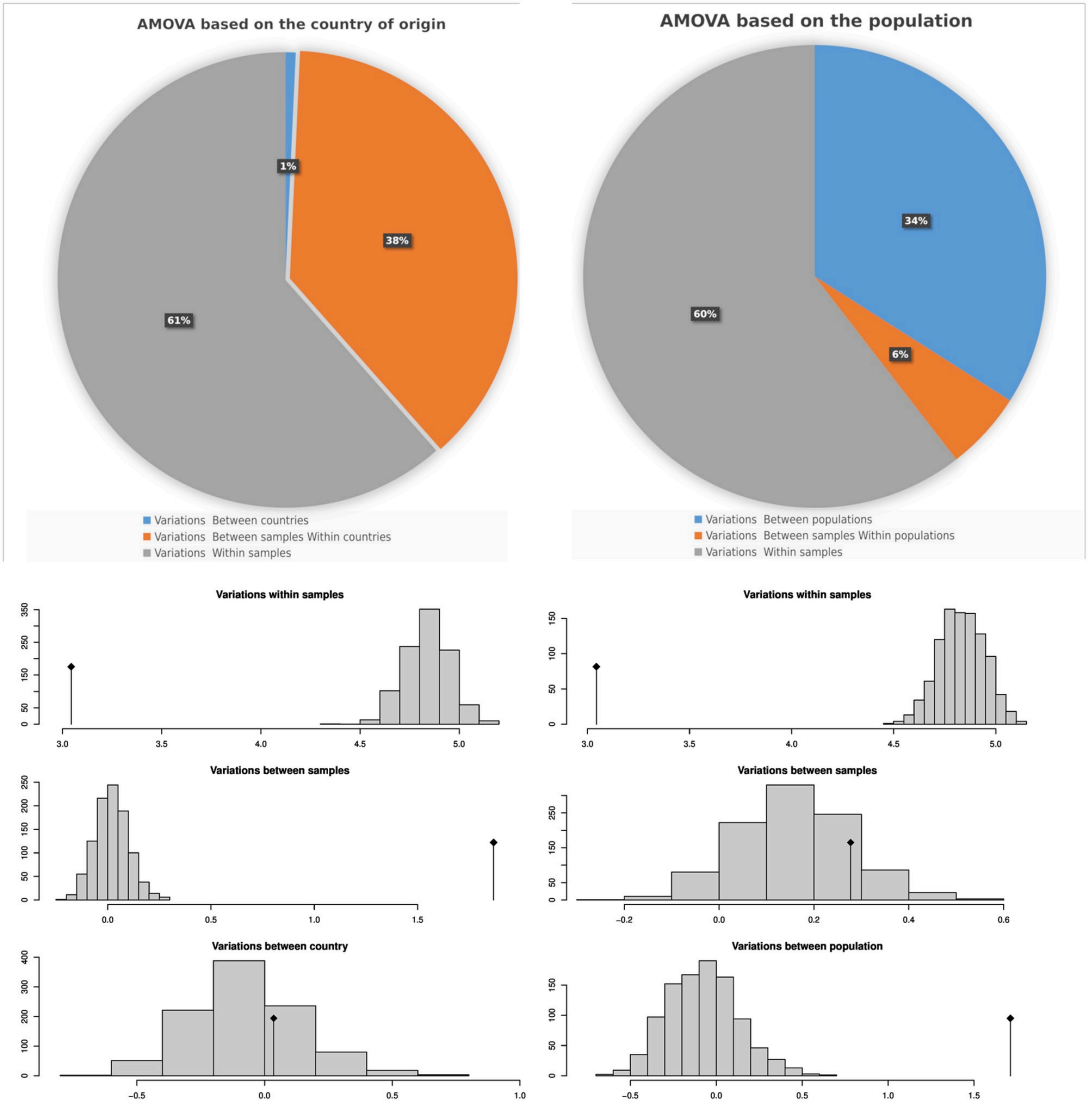
The analysis of molecular variance generated by genetic markers. AMOVA analysis of genetic variation among and within chickpea according to population, country of origin, as well as Monte-Carlo test results.

**Fig 6:**
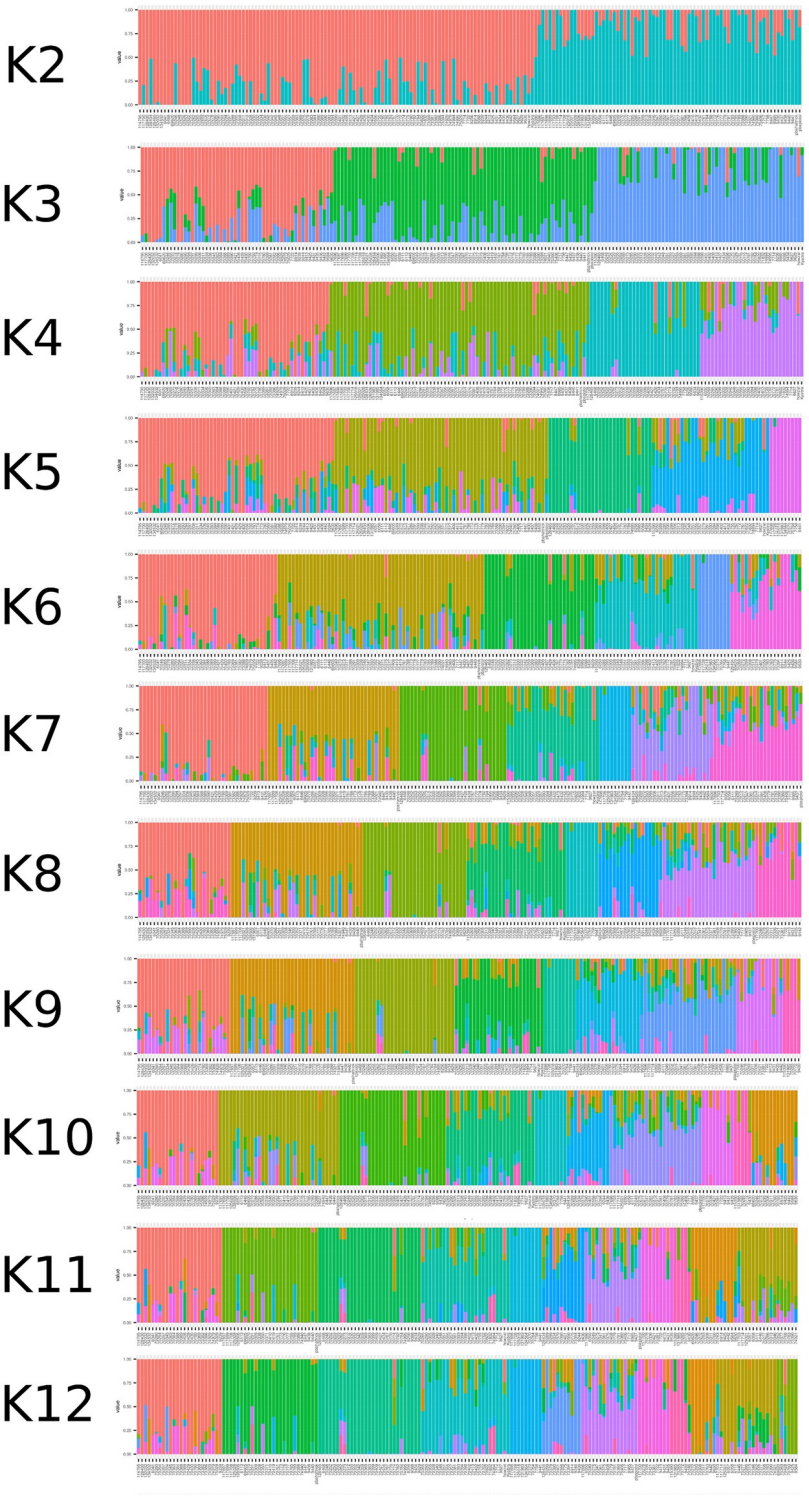
Chickpea population structure. Ancestry inference and genetic structure analysis of the chickpea populations based on the 1854 DArTs and SNPs markers conducted using ADMIXTURE software.

The genetic diversity of the chickpea population studied was investigated using various population statistics (Table 3). We calculated the rbarD [24] and the index of association (I^a^) [29] analysis to investigate the mode of reproduction and random matting. For a randomly mating population, the rbarD and (I^a^) expectations are zero. Clonal reproduction would be indicated by any significant deviation from the expected value of zero. The rbarD values ranged from 0.0005 (Nepal) to 0.124 (Azerbaijan), with a total of 0.03 (Table 3). In contrast, I^a^ ranged from 0.045 (Nepal) to 46.7 (Pakistan) and has shown a high value of I^a^ linked to chickpea sets of Pakistan, India, and Azerbaijan (Table 3). Additionally, we have calculated the Simpson’s Diversity Index (lambda) and heterozygosity (Hexp), which measures the population diversity. Lambda is a measure of diversity that considers the number of species present and their relative abundance [22]; while Hexp is a widely used metric for estimating expected heterozygosity, it underestimates true population diversity in samples with relatives [30]. The collective value of lambda was 0.99, where Pakistan and India demonstrated the highest lambda value (Table 3). In contrast, the chickpea sets showed the highest values of Hexp originated from India, Sudan, and Australia (Table 3). AMOVA revealed that geographical regions did not contribute significantly to the observed variation among chickpea accessions (1%), while the variations between and within accessions accounted for the remaining 38% and 61% (Fig 5). AMOVA, on the other hand, revealed that population information obtained from population structure analysis explained a significant portion of the variation observed between chickpea accessions (Fig 5). The population structure accounts for 34% of the variation observed between chickpea populations, while the variation between accessions accounted for 60% (Fig 5). The ADMIXTURE analysis revealed that the most probable sub-populations could be 12 as the average cross-validation (CV) error started to increase after this number (Fig 6; S1 Fig).

**Table 3:** The population statistics analysis, genotypic richness, and evenness using lambda Simpson’s Index [22], Hexp Nei’s unbiased gene diversity [23], the index of association (I^a^) [24], and the standardized index of association (rbarD).

Country	lambda	Hexp	I^a^	rbarD
The syrian Arab Republic	0	-	-	-
Pakistan	0.991	0.2485	46.1851	0.040047
India	0.973	0.3007	46.7998	0.044331
Australia	0.75	0.3898	15.4157	0.031538
Tunisia	0.5	0.1689	-	
Italy	0.667	0.1344	4.1964	0.076299
Turkey	0.75	0.2196	11.5709	0.054971
Russian Federation	0.667	0.1941	2.4573	0.015076
Azerbaijan	0.75	0.2832	35.8645	0.124714
Sudan	0.5	0.3364	-	
Ethiopia	0	-	-	-
Egypt	0.5	0.0541	-	
Nepal	0.667	0.1433	0.0449	0.000511
Total	0.995	0.2656	46.2768	0.03953

**Fig 7:**
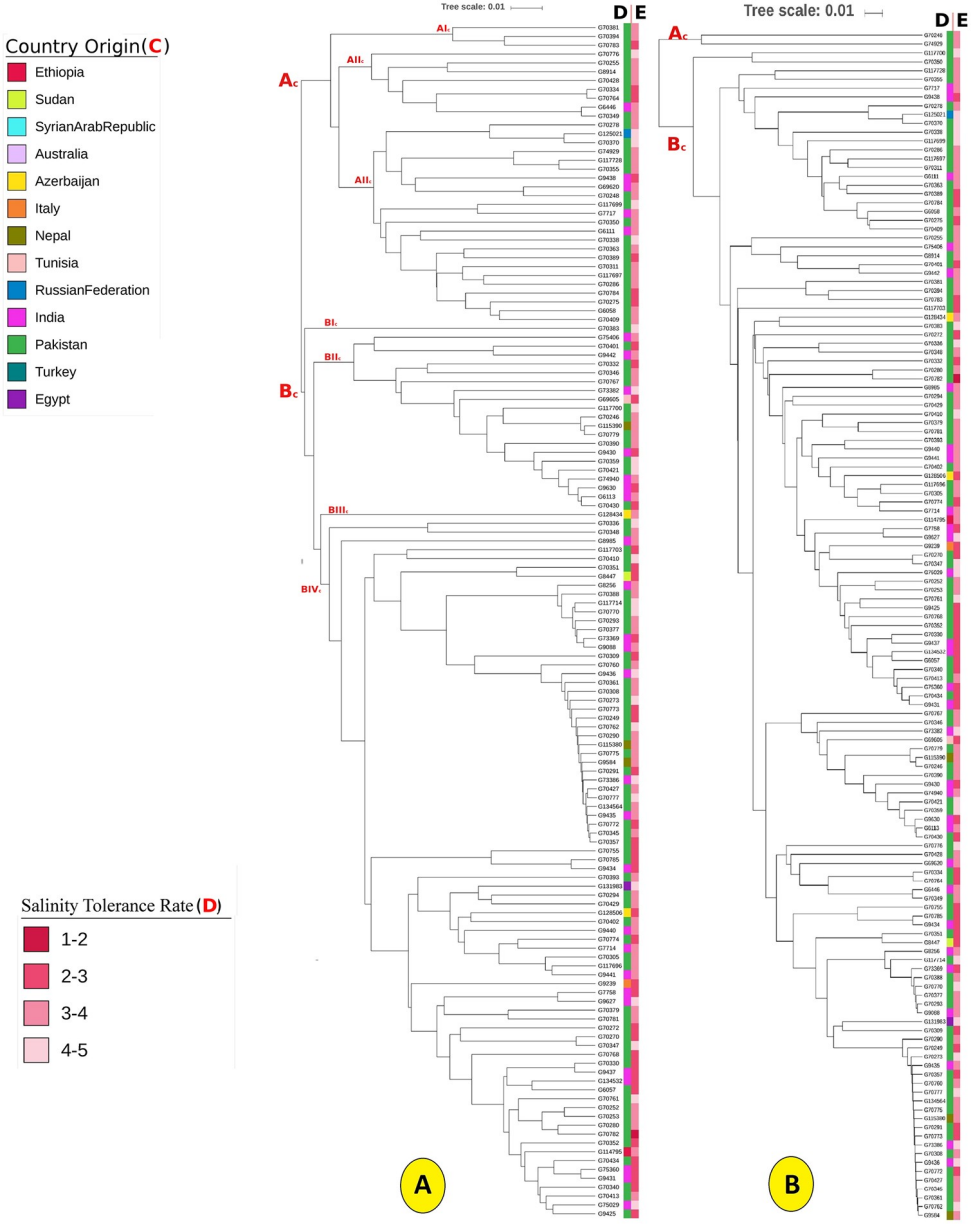
Phylogenetic trees conducted using genetic markers. The phylogenetic tree of chickpea genotypes based on SNP (A), and DArT (B) markers. The country origin (C) and the mean of phenotypic scale for salinity tolerance rate was assigned.

We used the SNP and DArT markers for phylogenetic tree construction. The maximum genetic dissimilarity between chickpea genotypes revealed by SNP markers was 0.53, the minimum was 0.004, and the median was 0.266. SNP-based phylogenetic tree clustered chickpea genotypes G70248 and G74929 in one cluster (Ac), while other genotypes were clustered in another major cluster. About 19 chickpea genotypes were extremely close to each other with moderately salinity tolerance and mainly from India and Pakistan (Fig 7). Depending on DArT and SNP markers, Phylogenetic analysis separated chickpea genotypes into two major clusters (Ac and Bc), where cluster “Bc” contains most chickpea genotypes. The Kinship analysis is an average dissimilarity between different genotypes. The genotypes originating from India or Italy have a higher dissimilarity than other genotypes (Fig 7).

### GWAS for salinity tolerance

**Fig 8:**
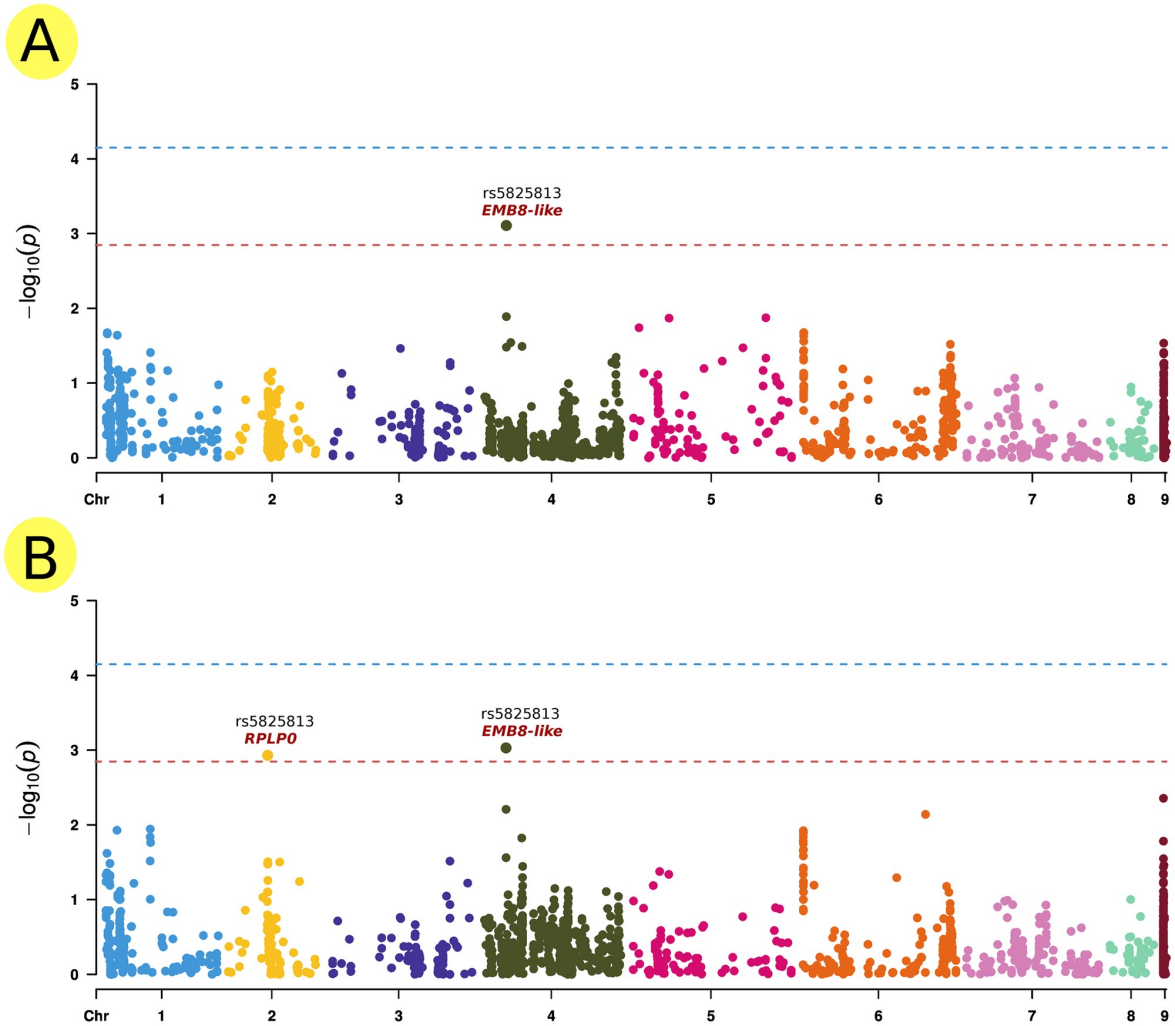
GWAS analysis results. Manhattan plots of highly associated haplotypes for salinity response trait under field and greenhouse conditions generated by mtGWAS analysis. A) Salinity-associated whole-genome markers identified on chromosome Ca4 at 10618070 bp (rs = 5825813) correlated with salinity tolerance in hydroponic and field systems, with a significant threshold for association at 3 x 10–3 located within the gene of *EMB8-like*. B) Salinity-associated identified on chromosome Ca2 at 30537619 bp (rs = 5825939) be correlated with salinity tolerance in hydroponic system located within the *RPLP0* gene.

**Fig 9:**
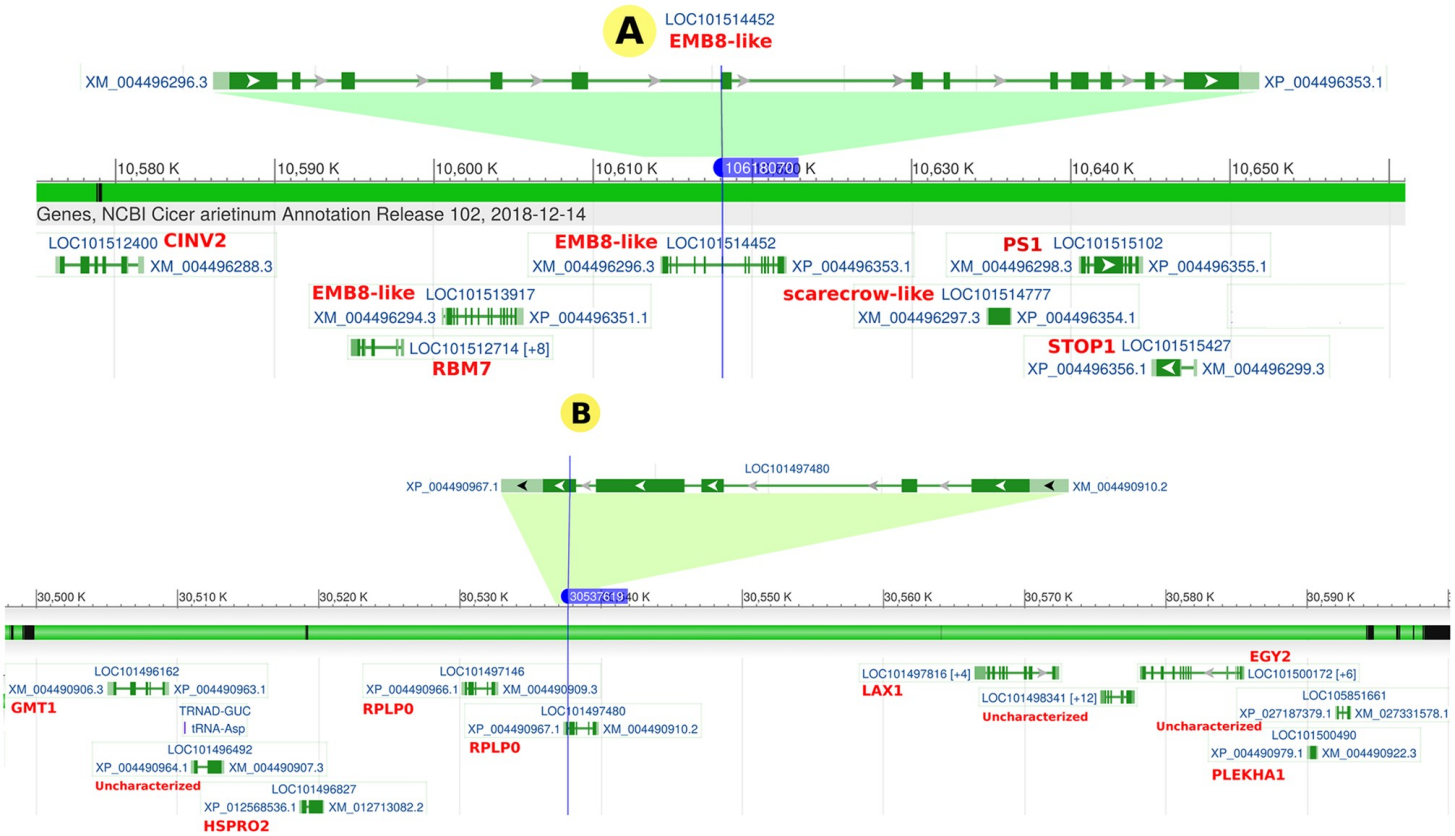
Salinity-tolerance associated genes. The location of the salinity-associated markers of 5825813 (**A**) and 5825939 (**B**) within chickpea genes.

The GEC method revealed that this set could be represented by 703.73 independent markers, which set the significance threshold for association at 7.11 x 10^-5^ and the suggestive threshold at 1.42 x 10^-3^. Multi-trait genome-wide association analysis (mtGWAS) detected one locus on chromosome Ca4 at 10618070 bp associated with salinity tolerance under both hydroponic and field systems with a significant threshold for association at 3 x 10^-3^ (rs = 5825813) and another locus-specific to the hydroponic system on chromosome Ca2 at 30537619 bp (rs = 5825939). The gene annotation analysis revealed that rs5825813 is located within the *Embryogenesis-associated protein (EMB8-like)* gene, while rs5825939 is located within the *Ribosomal Protein Large P0* (*RPLP0*) gene (Figs 8 and 9).

## Discussion

Chickpea is widely grown in West and Central Asia and Australia, where saline soils are abundant. There is little genetic variation among different genotypes, which is an obstacle to breeding for salinity tolerance. Additionally, providing salinity-tolerant chickpea genotypes could be a key to avoid adverse effects from other environmental stresses such as drought and heat [31]. In chickpea, despite several mapping studies, few studies have reported the presence of QTL for salinity tolerance, where a small number of salinity tolerance genes in chickpea have been reported [11, 32–34]. As a result, detecting markers associated with salinity tolerance and correlated with salinity-associated genes could aid marker-assisted selection. We have used a population set of 186 different chickpea genotypes collected from 28 provinces in 13 countries across the globe. The SNP and DArT markers demonstrated a moderate rate of PIC values from 0.01 to 0.375 and 0.0106 to 0.58, respectively. Other crop species, such as maize and soybean, have generated similar values of moderate PIC scores for genome-wide markers such as SNP and DArT [35, 36].

Sinai is a prosperous land for Egyptian agriculture; it has promising characteristics that, with proper management, should be able to fill a sizeable portion of the gap in local food production. Sinai has saline water and soil, which affect the growth of different crops [37]. For instance, several Egyptian wheat genotypes were studied under salinity stress, and genetic divergence was detected by evaluating genotypes under the Sinai environment using molecular and biochemical indicators [38, 39]. In our study, the phenotypic evaluation showed significant variations for salinity stress tolerance under saline conditions between various genotypes, indicating a potential of broad salinity tolerance within the global chickpea population. Seven salinity tolerant genotypes IGs (70782, 70430, 70764, 117703, 6057, 8447, and 70249) were identified with STR ≤ 2.5, of which 6 were from Pakistan and only one from Azerbaijan (Table 2). Previous studies tested several chickpea genotypes against varying saline levels. These studies found that salinity stress had a negative effect on chickpea genotypes during early growth stages, with the magnitude of responses varying across genotypes, indicating various levels of genotype-environment interactions [40]. Comparable results were reported by Baloch et al., in wheat [41].

Studying genetic diversity in chickpea should help us better understand the natural variation of phenotypic traits and their genetic background. To investigate the genetic diversity in the chickpea set under study, we used various population statistics. The PCA analysis was used to deduce genetic variation revealed by genetic markers. It was also used to investigate the relationship between geographic and genetic diversity (Fig 4B). The PCA revealed that most of the chickpea genotypes studied have little genetic variation between geographical groups. In contrast, some chickpea genotypes, particularly those from Pakistan and India, have a high rate of genetic variation. According to the squared cosine value (*cos^2^*), several chickpea genotypes strongly contributed to the genetic variation depicted by PCA (Fig 4A). Moreover, the genetic richness and variation among genotypes from Pakistan and India are high compared to other geographical populations, according to population statistics indices such as rbarD, I, lambda, and Hexp. Due to their similar environments and agro-ecological zones, Pakistani and Indian genotypes share genetic variation. The AMOVA did not reveal a significant variation between countries (1%), suggesting that geographic regions do not affect chickpea diversity. In contrast, the genetic variation found between the chickpea populations was greater than between countries (Fig 5). The ADMIXTURE analysis revealed that the most probable sub-populations could be 12 groups (Fig 6), which may reflect the difference of origin in our chickpea population (S1 Table). Basu *et al.* [42] used genome-wide SNPs polymorphism to study the genetic diversity of a selected population of Desi and Kabuli chickpea genotypes. Their results demonstrated that geographic origin and the adaptive environment affected the clustering of genotypes into a specific population group more than their known pedigree and parentage.

In our study, we used genome-wide DArTseq-based SNP markers to study the population structure of 186 chickpea genotypes. They discovered a high genetic diversity between genotype pairs, suggesting that the chickpea genotypes studied have a diverse genetic lineage [43]. The phylogenetic trees showed that the salinity tolerance rate has a minor effect on the genetic structure of the studied chickpea genotypes.

Previous research on the genetic structure of chickpea salinity tolerance revealed several key factors that may control the biological responses of chickpea. Pushpavalli *et al.* [11] found 48 putative candidate genes responsive to salinity stress on CaLG05 and CaLG07, which are in a distance of 11.1 Mb and 8.2 Mb, respectively. Most of the genes were involved in achieving osmoregulation under stress conditions. Soren *et al.* [34] have recently identified a group of candidate genes linked to chickpea salinity tolerance. They found significant QTLs for yield and yield component traits under salinity stress on CaLG03 (3.3 Mb region) and CaLG06 (0.1 Mb region). They also found several genes that may be important for salinity tolerance in these two clusters. These genes include calcium-dependent protein kinases, histidine kinases, cation proton antiporter, and WRKY and MYB transcription factors. In the present study, mtGWAS analysis revealed one locus on chromosome Ca4 at 10618070 bp to be associated with salinity tolerance under both hydroponic and field systems with a significant threshold for association at 3 x 10^-3^ (rs = 5825813) and another locus-specific to the hydroponic system on chromosome Ca2 at 30537619 bp (rs = 5825939) (Fig 8).

The gene annotation analysis revealed that rs5825813 is located within the gene of *Embryogenesis-associated protein (EMB8-like)*, while rs5825939 is located within *Ribosomal Protein Large P0* (*RPLP0*) gene (Fig 9). Embryogenesis is a critical developmental phase in the life cycle of plants that spans the transformation from fertilized egg to mature embryo generation [44]. The correlation between genetic variations within a gene member of the Embryogenesis-associated gene family and salinity tolerance in chickpea is not surprising. Genes of this family play a critical role in biotic and abiotic tolerance in various plant species [45]. Unfortunately, the role of *EMB8* in the embryogenesis process is not yet explained, although its potential role in other plant species such as cotton has been reported [46]. Conversely, the RPLP0 gene belongs to the acidic ribosomal P proteins family, which is considered to be directly involved in interactions with elongation factors (EF1) during protein synthesis. Moreover, there is evidence that P proteins are involved in DNA repair and transcription [47]. In barely, an increase in all ribosomal proteins was observed during salinity stress, indicating resistance to the inhibitory effect of NaCl on protein biosynthesis [48]. However, these annotated genes might not be responsible for salinity tolerance given the high LD existed in the studied population (≃0.9 Mb). The two associated SNPs might have high LD with neighboring genes within the same LD block responsible for salinity tolerance.

## Supporting information

S1 Table The information of the chickpea subset. The chickpea subset was selected from ICARDA’s genebank using the FIGS strategy.
(XLS)

S2 Table Genetic marker and phenotypic data. The genetic variation of SNP and DArT markers evaluated across a population set of 186 different chickpea genotypes collected from 28 provinces in 13 countries across the globe. The genetic markers were filtered based on MAF and missing values.
(XLSX)

S1 Fig Cross-validation (CV) error rates. The cross-validation (CV) error rates of ADMIXTURE results.
(TIF)
