# Supplementary figures and images for: Genome-wide association analysis of chickpea germplasms differing for salinity tolerance based on DArTseq markers
Source: PLoS One. 2021 Dec 1;16(12):e0260709. doi: 10.1371/journal.pone.0260709 (PMC8635330; doi:10.1371/journal.pone.0260709)

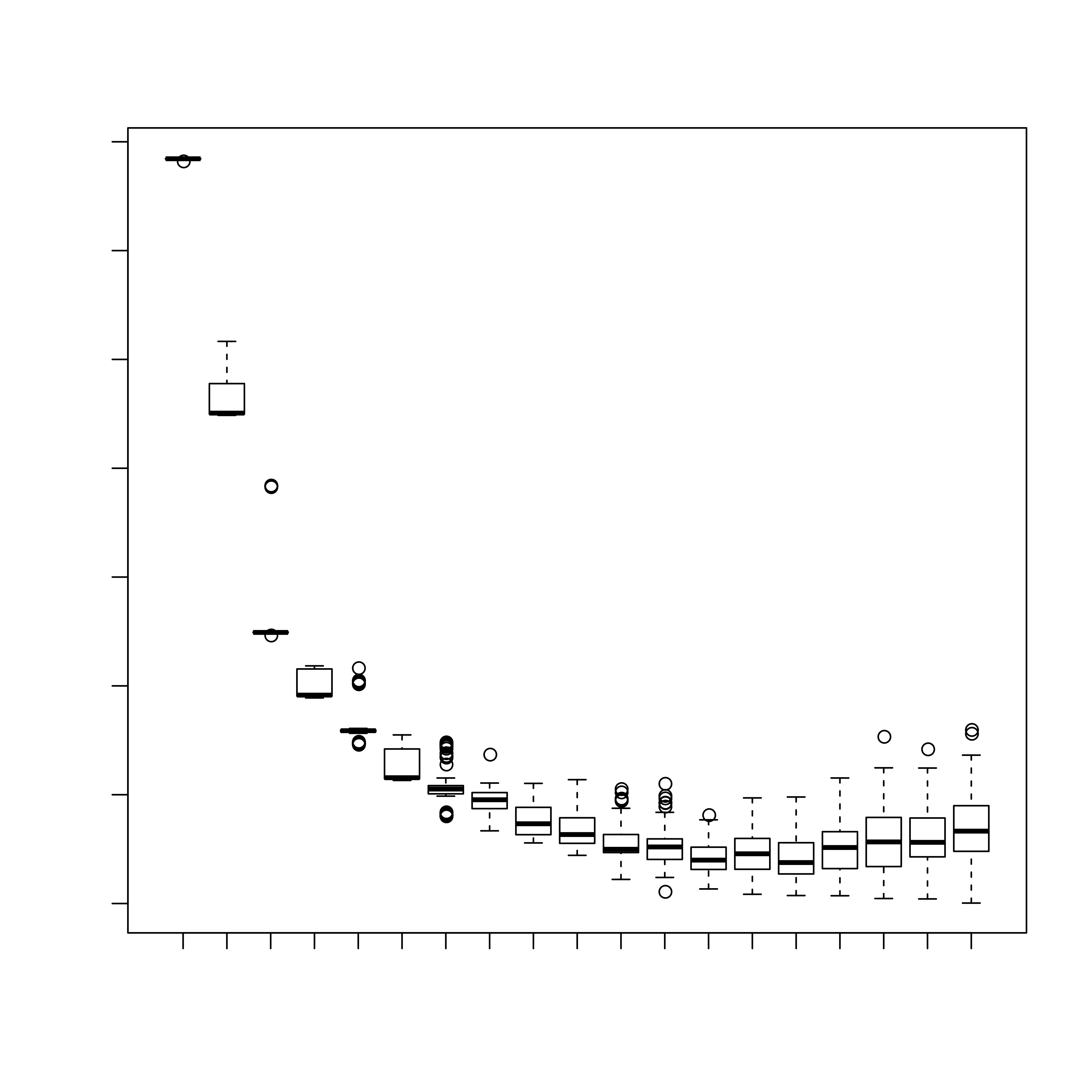

Supplement: S1 Fig — The cross-validation (CV) error rates of ADMIXTURE results. (TIF) [file pone.0260709.s003.tif]
